# The multimodal psychotherapy of the anxiety disorders patients

**DOI:** 10.1192/j.eurpsy.2024.882

**Published:** 2024-08-27

**Authors:** V. Mishyiev, B. Mykhaylov, Y. Grynevych, V. Omelyanovich

**Affiliations:** ^1^ Shupyk National Healthcare University of Ukraine; ^2^Bogomolets National Medical University, Kyiv, Ukraine

## Abstract

**Introduction:**

Anxiety disorders are a common type of mental pathology with severe social and medical consequences in the lives of people suffering from them. General population studies indicate their prevalence ranges from 1.7% to 4.7% of the population. According to data from a US national study among the population aged 15 to 54 years, only 2.7% and 4.7%, respectively, suffered from panic disorder, one of the common types of anxiety disorders, during their lifetime. At the same time, the features of the emotional structure of anxiety disorders and the effectiveness of their psychotherapy among the population of low-income countries, especially in countries in a situation of prolonged bloody war and environmental disaster, remain poorly studied.

**Objectives:**

The purpose of the study was to identify the features of the emotional symptomatic structure of anxiety disorder and evaluate the effectiveness of their psychotherapeutic correction. For this purpose, 180 patients with anxiety disorders who were hospitalized in Ukraine (during the period 2022 - 2023) were examined.

**Methods:**

The basic method was group psychotherapy with elements of rational, positive, suggestive and family psychotherapy. Regarding emotional disorders, cognitive behavioural therapy (CBT) was used for phobic-depressive and anxiety-depressive syndromes.

**Results:**

Most patients experienced a decrease in the level of general anxiety and internal anxiety. Almost no spontaneous occurrence of fear was observed. During active interviewing, patients stated that their previous anxieties and fears had lost their relevance and acquired clear emotional overtones. There was also a significant decrease in the symptoms of the depressive cycle, and patients began to feel joy and optimism.

**Conclusions:**

To correct emotional dysfunction in patients with episodic paroxysmal disorders, generalized anxiety disorders and mixed anxiety-depressive disorders, it is optimal to use a system of psychotherapeutic correction built on stepwise and multimodal principles.

**Disclosure of Interest:**

None Declared

